# Citation classics in general medical journals: assessing the quality of evidence; a systematic review 

**Published:** 2020

**Authors:** Suhaib JS. Ahmad, Ahmed R. Ahmed, Karl Friedrich Kowalewski, Felix Nickel, Kamran Rostami, Claire J Stocker, Sherif M. Hakky, Rami Archid, Douglas McWhinnie, Ata Mohajer-Bastami, Dionysis Skiadopoulos Seimenis, Sami Ahmad, Sami Mansour, Mohamed H. Ahmed, Dushyant Mital, Aristomenis K. Exadaktylos

**Affiliations:** 1 *Undergraduate Medical School, University of Buckingham, Buckingham, UK*; 2 *Department of Bariatric and Metabolic Surgery, Imperial College London, London, UK*; 3 *Department of General, Visceral and Transplantation Surgery, University of Heidelberg, Heidelberg, Germany *; 4 *Department of Gastroenterology, Palmerston North Hospital, New Zealand v*; 5 *Kasr Al Ainy School of Medicine, University of Cairo, Egypt *; 6 *Department of General, Visceral and Transplant Surgery, Eberhard-Karls- University Hospital Tuebingen, Tuebingen, Germany*; 7 *Department of Surgery, Milton Keynes University Hospital, Milton Keynes, UK *; 8 *Department of Surgery, Chelsea and Westminster Hospital, London, UK *; 9 *Istishari Hospital, Amman, Jordan *; 10 *Department of Medicine and HIV Metabolic Clinic, Milton Keynes University Hospital NHS Foundation Trust, Milton Keynes, UK *; 11 *Departmernt of HIV and Blood Borne Viruses, Milton Keynes University Hospital, Milton Keynes, UK *; 12 *Department of Emergency Medicine, Inselspital, University Hospital of Bern, Bern, Switzerland *

**Keywords:** Most-cited articles, Bibliometrics, Level of evidence, Citation classics, General medical journals, Article quality

## Abstract

**Aim:**

This review provides a comprehensive overview of more than 100 of the most cited studies in general medical journals and evaluates whether citations predict the quality of a scientific article.

**Background:**

The number of citations is commonly used as a measure of the quality and impact of a scientific article. However, it is often criticised that the number of citations is in fact a poor indicator of the true quality, as it can be influenced by different factors such as current trends.

**Methods:**

This review was conducted in line with the PRISMA guidelines. The Journal Citation Report (JCR) within Incites allowed the evaluation and comparison of articles, published in general medical journals, using far-reaching citation data drawn from scholarly and technical journals and conference proceedings. All steps of the review were performed in duplicate and conflicts were resolved through consensus.

**Results:**

The 100 most cited articles published from 1963 until the end of 2018 were identified. The number of citations ranged from 4012 to 31853. Most of the articles were published in the 2000’s, followed by the 1990’s, 1980’s, 1970’s and 1960’s, respectively. All of the articles were published in five journals. There were 50 studies at level II, 28 at level V, 10 at level IV, 7 at level III, and 5 at Level I.

**Conclusion:**

This systematic review provides an overview of the most cited articles, published in general medical journals. The number of citations provides an indication of the quality of evidence. However, researchers and clinicians should use standardized assessment tools rather than solely rely on the number of citations in order to judge the quality of published articles.

## Introduction

 The term Evidence-Based Medicine (EBM) was first coined by Guyatt, in 1991. It refers to the meticulous, explicit and prudent use of clinical expertise, patients’ values and the best available scientific evidence in making decisions on the care of individual patients (1). 

A cornerstone of EBM is the hierarchical system of classifying evidence. The echelon system, known as the levels of evidence (LOE), was first described in a report by the Canadian Task Force in 1979 (2). The purpose of the report was to develop recommendations on the periodic health examination based on evidence available in the medical literature. The quality of the evidence was determined by the degree to which it reflected the true theoretical effect of the intervention. The LOE system was further defined by Sackett in 1989 (3). The early hierarchy systems considered systematic reviews and randomised controlled trials (RCT’s) to have the highest LOE, while case reports and expert opinion had the lowest LOE (4). This is because RCT’s are designed to minimise bias and systematic error while on the other hand, expert opinion is frequently biased by the author`s experience and the lack of control. 

Over the past 20 years, the volume of published scientific literature has increased exponentially, and identifying relevant information has become a complex task for the individual investigator (5). Thus, researchers are encouraged to endorse the core principles of the hierarchy of evidence to answer definitive research questions. 

A citation is the acknowledgment one gives to a published or unpublished source. Citation count is regarded as a useful tool in obtaining a quantitative measure of the utilisation and contribution of a particular published paper. It also reflects the impact of the author’s intellectual capability (6). However, whether the number of citations echoes the methodological quality remains an open question. Recently, several attempts have been made to identify and analyse highly cited articles, allowing the reader to understand their quality and characteristics (7-9).

This bibliometric study identifies citation classics, published in general medical journals, and applies the empirical data to establish a quantitative assessment of the academic output, and to demonstrate the extent to which the number of citations can predict quality. This will allow us to reveal whether the number of citations can be utilised as a requirement of objective criteria for faculty hiring as well as performance evaluation. Furthermore, controversies concerning technical limitations of citations, database selectivity, time and discipline-related bias, publication type bias, authorships merits, and motivations for citing are addressed. 

## Methods

*The reporting of this systematic review conforms to the Preferred Reporting Items for Systematic Reviews and Meta-analysis (PRISMA) guidelines (10).

**Information sources**

The Web of Science was used to provide comprehensive citation data for articles, published in general medical journals. The Web of Science allowed the following databases to be identified: Medline, Web of Science Core Collection, BIOSIS previews, and SciELo Citation index. 

The Journal Citation Report (JCR), within the Web of Science, allowed the evaluation and comparison of articles, published in broad-ranging medical journals, using far-reaching citation data drawn from scholarly and technical journals and conference proceedings. The JCR allowed the following information to be extracted: 

*Journal-related data*

1-Bibliographic information of publisher, title abbreviation, language and ISSN.

2-Subject Categories.

*Basic citation-related data*

1-The number of articles published during that year and the number of citations that the articles have accrued. 

*Detailed citation-related data*

1-The number of times an article was cited, by later published articles, during the year. 

2-The number of citations made from articles published in the journal, during each of the most recent 10 years. 

3-The number of times articles, published in a specific journal, were cited by other journals, during each of the most recent 10 years. 

*Several measures can be derived from these data including the impact factor, immediacy index, quartiles & JIF percentiles, cited & citing half-life.

Categories by Rank within the JCR was used to list all subject categories, ranked by the number of journals. Journals within the subject category `Medicine, General & Internal` were included and the data through which that year’s calculated metric was displayed. For overall systematic review credibility, peer-reviewed open access journals were included in the study, with no language restriction. The search was conducted on the 16/12/2018 by two reviewers, with experience in bibliometrics, who independently reviewed the journals, articles, and abstracted data. They also resolved any arising disagreements through consensus. 

**Eligibility criteria**

**Figure 1:**
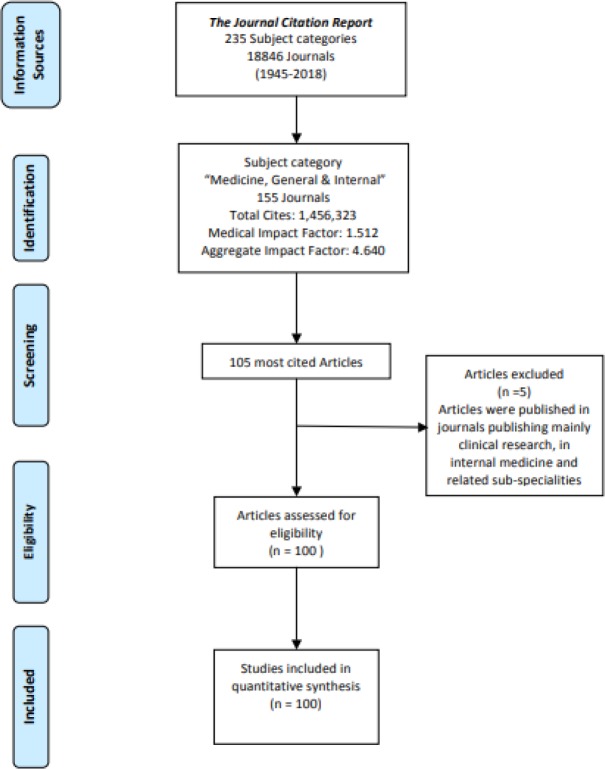
Methods’ Flowchart

The 100 most-frequently cited articles published in journals, within the subject category `Medicine, General & Internal`, were identified. Articles were included in data extraction if they were published in peer-reviewed journals, covering the full spectrum of the medical sciences. Journals publishing mainly clinical research, in internal medicine and related sub-specialities, were excluded from the study (Figure 1).

**Data collection process**

The 100 most cited articles, published in general medical journals, were reviewed with the following information retrieved and compiled: (1) name of the Journal; (2) article title; (3) article’s age; (4) citation number; (5) citation density (mean number of citations per year=total number of citations/years since publication of the article); (6) journal’s quartile; (7) journal’s country of origin; (8) authorship; (9) first author’s institution/country of origin and continent; (10) citation number; (11) field of medicine (alternative medicine, angiology, biochemistry, cardiology, cytogenetics, endocrinology, geriatrics, hepatology, infectious disease, intensive care, medical statistics, nephrology, neurology, oncology, psychiatry, public health, pulmonology, quality of reporting); (12) article classification (Original, review, experimental) ; (13) article methodology (systematic review, randomised controlled trial, cohort, case control, case series, cross-sectional study, clinical consensus, expert opinion, laboratory study); (14) level of evidence.

*The institution and country of origin were defined based on the affiliation provided for the first author. In the case of group authorship, the affiliation was regarded that of the corresponding author. In the case of papers accepted in more than one journal, only the top cited paper was included in the study. The Oxford Centre for Evidence‐Based Medicine (OCEBM) classification was used to analyse the level of evidence of articles (11, 12).

**Levels of evidence**

Level 1: systematic review of randomised-controlled trials/ systematic review of inception cohort studies/ systematic review of nested case-control studies/ systematic review of cross-sectional studies with consistently applied reference standard and blinding/ local and current random sample surveys or censuses.

Level 2: systematic review of surveys that allow matching local circumstances/ cross-sectional studies with consistently applied reference standard and blinding/ inception cohort studies/ randomized controlled trials/ observation study with dramatic effects.

Level 3: Local non-random sample/ non-consecutive studies/ studies without consistently applied reference standards/ cohort study or control arm of randomized trials/ non-randomized controlled cohort/follow up studies.

Level 4: case-series/ case-control/ poor or non-independent reference standard/ poor quality prognostic cohort study/ historically controlled studies.

Level 5: mechanism-based reasoning/ expert opinion (11, 12).

**Data synthesis and analysis**

Statistical analysis was performed using only the data from studies in the extracted subset. The tidyverse collection of R programming language and its libraries, version 3.5, was used to implement a wide variety of statistical and graphical techniques. The Shapiro-Wilk test was applied to estimate the variance of the sample. The Pearson R correlation was employed to measure the degree of the relationship amongst linear related variables. The non-parametric tests, Kendall rank correlation and Kruskal-Wallis test, were used to measure the strength of interdependence between variables and to assess significant differences on a continuous dependent variable through a categorical independent variable respectively. 

The Pearson R and Kendall rank correlations results were expressed as a range between -1 and 1, with -1 being a strong negative correlation and 1 a strong positive correlation. Probability values were two-tailored and the threshold for significance was set at p<0.05. 

**Patient and Public Involvement:**

Patients and public were not involved in this study. 

## Results

**Citation count and density**

The total number of citations ranged from 4021 to 31853. The mean number of citations stood at 6179 (normally distributed data). Seven articles received over 10000 citations, and more than half of the articles had over 5000 citations. The citation density (the mean number of citations per year=total number of citations/years since publication of the article) varied from 112 to 2258. The median citation density was 314 (non-normally distributed data) and over half of the articles had a density of over 300, as shown in Table 1.

**Year of publication**

The hundred most cited articles were published between 1963 and 2014. The decade witnessing the highest number of articles was the 2000’s (58 articles), followed by the 1990’s (31 articles), the 1980’s (5 articles), the 1970’s (4 articles) and the 1960’s (2 articles). The decade 2000’s contributed to over half (339243 citations/55%) of the overall number of citations, followed by the 1990’s (189826 citations/31%), the 1980’s (53415 citations/8%), 1970’s (25193 citations/4%), and 1960’s (10200 citations/2%). The single year with the highest number of citations was 1968 while the lowest was 1963. The citation density ranged from 185 in the 1960’s to peak at 28673 in the 2000’s. The articles published in 2009 had the maximum density of 2258, while the articles published in 1963 had the minimum at 73 (Table 1).

**Journals publishing the citation classics**

The citation classics were published in 5 different journals; these were predominantly comprehensive medical journals, led by New England Journal of Medicine with 57 articles, followed by Lancet with 21 articles, Journal of the American Medical Association with 17 articles, British Medical Journal with 4 articles and Plos Medicine with 1 article. The impact factor of the academic journals ranged from 11.675 for Plos Medicine to 79.260 for New England Journal of Medicine. Further, 95% of the articles were published in journals with impact factor higher than 47.661. Table 2 lists the journals in which the citation classics were published in. 

**Authorship, country of origin and institutions**

**Figure 2:**
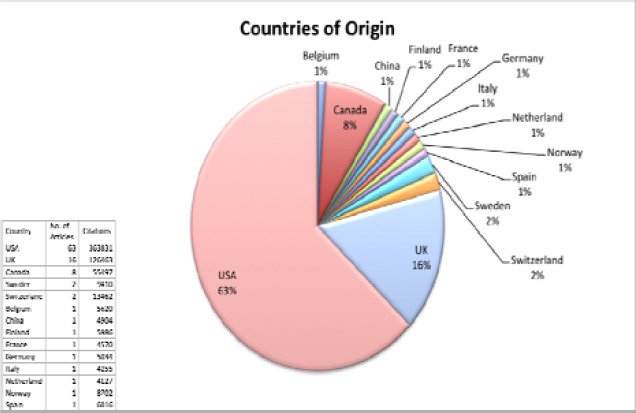
A pie chart depicting information about the countries of origin, of the hundred most cited articles

The majority of the citation classics were produced by three or more authors (85%). With regards to individual contributions, Bland JM was the author of the most cited publication, with 31853 citations. The second most cited publication, with 20319 citations, was published by Moher D, whose name appeared in three articles within the 100 most cited list. Randle PJ authored the publication with the minimum citation number (4021 citations). Memish ZA contributed to three of the 100 most cited articles (14614 citations), followed by Altman DG (36109 citations), Ross R (21335 citations), National Cancer Institute of Canada Clinical Trials Group (13218 citations), Folkman J (12300) and Flegal KM (10395 citations), each of whom contributed to 2 articles respectively. The citation classics originated from 14 different countries. A total of 63 articles were published by authors from the USA. The United Kingdom was the second most productive country with 16 articles published, followed by Canada with 8 articles (Figure 2). 

Articles originating from the USA had the largest total number of citations (363831), followed by the UK (126463) and Canada (55497). 

**Table 1:** Citation classics in general medical journals

First author	Citations	Density	Field	Title
Bland JM	31853	995	Medical Statistics	Statistical methods for assessing agreement between two methods of clinical measurement.
Moher D	20319	2258	Quality of Reporting	Preferred reporting items for systematic reviews and meta-analyses: the PRISMA statement.
Egger M	19428	925	Quality of Reporting	Bias in meta-analysis detected by a simple, graphical test.
Ross R	16559	872	Cardiology	Atherosclerosis--an inflammatory disease.
Chobanian AV	12581	839	Cardiology	The Seventh Report of the Joint National Committee on Prevention, Detection, Evaluation, and Treatment of High Blood Pressure: the JNC 7 report.
Expert Panel on Detection, Evaluation, and Treatment of High Blood Cholesterol in Adults.	10919	642	Cardiology	Executive Summary of The Third Report of The National Cholesterol Education Program (NCEP) Expert Panel on Detection, Evaluation, And Treatment of High Blood Cholesterol In Adults (Adult Treatment Panel III).
Diabetes Control and Complications Trial Research Group,	10843	434	Endocrinology	The effect of intensive treatment of diabetes on the development and progression of long-term complications in insulin-dependent diabetes mellitus.
Rossouw JE	9543	596	Endocrinology	Risks and benefits of estrogen plus progestin in healthy postmenopausal women: principal results From the Women's Health Initiative randomized controlled trial.
Diabetes Prevention Program Research Group	9477	592	Endocrinology	Reduction in the incidence of type 2 diabetes with lifestyle intervention or metformin.
UK Prospective Diabetes Study (UKPDS) Group	9149	457	Endocrinology	Intensive blood-glucose control with sulphonylureas or insulin compared with conventional treatment and risk of complications in patients with type 2 diabetes (UKPDS 33). UK Prospective Diabetes Study (UKPDS) Group.
Stupp R	8968	690	Oncology	Radiotherapy plus concomitant and adjuvant temozolomide for glioblastoma.
Stroup DF	8851	492	Quality of Reporting	Meta-analysis of observational studies in epidemiology: a proposal for reporting. Meta-analysis Of Observational Studies in Epidemiology (MOOSE) group.
Scandinavian Simvastatin Survival Study Group	8702	363	Cardiology	Randomised trial of cholesterol lowering in 4444 patients with coronary heart disease: the Scandinavian Simvastatin Survival Study (4S).
Lynch TJ	7875	563	Oncology	Activating mutations in the epidermal growth factor receptor underlying responsiveness of non-small-cell lung cancer to gefitinib..
Teasdale G	7571	172	Neurology	Assessment of coma and impaired consciousness. A practical scale.
Folkman J	7375	157	Oncology	Tumor angiogenesis: therapeutic implications.
Hurwitz H	7348	525	Oncology	Bevacizumab plus irinotecan, fluorouracil, and leucovorin for metastatic colorectal cancer.
Holick MF	7302	664	Endocrinology	Vitamin D deficiency.
Slamon DJ	7002	412	Oncology	Use of chemotherapy plus a monoclonal antibody against HER2 for metastatic breast cancer that overexpresses HER2.
Palella FJ Jr	6721	336	Infectious Diseases	Declining morbidity and mortality among patients with advanced human immunodeficiency virus infection. HIV Outpatient Study Investigators.
Hodi FS	6695	837	Oncology	Improved survival with ipilimumab in patients with metastatic melanoma.
Go AS	6456	461	Nephrology	Chronic kidney disease and the risks of death, cardiovascular events, and hospitalization.
Young T	6455	258	Pulmonology	The occurrence of sleep-disordered breathing among middle-aged adults.
Steinberg D	6289	217	Cardiology	Beyond cholesterol. Modifications of low-density lipoprotein that increase its atherogenicity.
Heart Outcomes Prevention Evaluation Study Investigators	6185	344	Cardiology	Effects of an angiotensin-converting-enzyme inhibitor, ramipril, on cardiovascular events in high-risk patients.
Katz S	6179	112	Geriatrics	Studies of illness in The aged. The Index of ADL: A Standardized measure of biological and psychologial Function.
Shepherd J	6175	268	Cardiology	Prevention of coronary heart disease with pravastatin in men with hypercholesterolemia. West of Scotland Coronary Prevention Study Group.
Ogden CL	6104	509	Public Health	Prevalence of overweight and obesity in the United States, 1999-2004.
Rivers E	6069	357	Intensive Care	Early goal-directed therapy in the treatment of severe sepsis and septic shock.
Llovet JM	6016	602	Oncology	Sorafenib in advanced hepatocellular carcinoma.
Tuomilehto J	5986	352	Endocrinology	Prevention of type 2 diabetes mellitus by changes in lifestyle among subjects with impaired glucose tolerance.
Sacks FM	5719	260	Cardiology	The effect of pravastatin on coronary events after myocardial infarction in patients with average cholesterol levels. Cholesterol and Recurrent Events Trial investigators.
Connolly SJ	5716	635	Cardiology	Dabigatran versus warfarin in patients with atrial fibrillation.
Yusuf S	5706	408	Cardiology	Effect of potentially modifiable risk factors associated with myocardial infarction in 52 countries (the INTERHEART study): case-control study.
van den Berghe G	5620	331	Intensive Care	Intensive insulin therapy in critically ill patients.
National Institute of Neurological Disorders and Stroke rt-PA Stroke Study Group	5610	244	Neurology	Tissue plasminogen activator for acute ischemic stroke.
Lozano R	5503	917	Public Health	Global and regional mortality from 235 causes of death for 20 age groups in 1990 and 2010: a systematic analysis for the Global Burden of Disease Study 2010.
Vogelstein B	5379	179	Oncology	Genetic alterations during colorectal-tumor development.
Pitt B	5377	283	Cardiology	The effect of spironolactone on morbidity and mortality in patients with severe heart failure.
Prospective Studies Collaboration	5375	336	Cardiology	Age-specific relevance of usual blood pressure to vascular mortality: a meta-analysis of individual data for one million adults in 61 prospective studies.
Heart Protection Study Collaborative Group	5308	332	Cardiology	MRC/BHF Heart Protection Study of cholesterol lowering with simvastatin in 20,536 high-risk individuals: a randomised placebo-controlled trial.
Hansson GK	5286	407	Cardiology	Inflammation, atherosclerosis, and coronary artery disease.
Topalian SL	5272	879	Oncology	Safety, activity, and immune correlates of anti-PD-1 antibody in cancer.
Seabright M	5186	110	Cytogenetics	A rapid banding technique for human chromosomes.
McCord JM	5118	155	Angiology	Oxygen-derived free radicals in postischemic tissue injury.
Moncada S	5102	204	Biochemistry	The L-arginine-nitric oxide pathway.
Lim SS	5089	848	Public Health	A comparative risk assessment of burden of disease and injury attributable to 67 risk factors and risk factor clusters in 21 regions, 1990-2010: a systematic analysis for the Global Burden of Disease Study 2010.
Jennett B	5061	118	Neurology	Assessment of outcome after severe brain damage.
Fried MW	5038	315	Hepatology	Peginterferon alfa-2a plus ribavirin for chronic hepatitis C virus infection.
Manns MP	5034	296	Hepatology	Peginterferon alfa-2b plus ribavirin compared with interferon alfa-2b plus ribavirin for initial treatment of chronic hepatitis C: a randomised trial.
Higgins JP	5027	718	Quality of Reporting	The Cochrane Collaboration's tool for assessing risk of bias in randomised trials.
Weidner N	4925	182	Oncology	Tumor angiogenesis and metastasis--correlation in invasive breast carcinoma.
Mok TS	4904	545	Oncology	Gefitinib or carboplatin-paclitaxel in pulmonary adenocarcinoma.
Acute Respiratory Distress Syndrome Network	4870	271	Pulmonology	Ventilation with lower tidal volumes as compared with traditional tidal volumes for acute lung injury and the acute respiratory distress syndrome.
Dockery DW	4806	192	Public Health	An association between air pollution and mortality in six U.S. cities.
Ross R	4776	149	Cardiology	The pathogenesis of atherosclerosis--an update.
Pate RR	4768	207	Public Health	Physical activity and public health. A recommendation from the Centers for Disease Control and Prevention and the American College of Sports Medicine.
Brenner DJ	4713	428	Radiology	Computed tomography--an increasing source of radiation exposure
Considine RV	4690	213	Biochemistry	Serum immunoreactive-leptin concentrations in normal-weight and obese humans.
Pfeffer MA	4640	178	Cardiology	Effect of captopril on mortality and morbidity in patients with left ventricular dysfunction after myocardial infarction. Results of the survival and ventricular enlargement trial. The SAVE Investigators.
Kessler RC	4608	304	Psychiatry	The epidemiology of major depressive disorder: results from the National Comorbidity Survey Replication (NCS-R). JAMA. 2003 Jun 18;289(23):3095-105.
Guyatt GH	4574	457	Quality of Reporting	GRADE: an emerging consensus on rating quality of evidence and strength of recommendations.
Haïssaguerre M	4570	229	Cardiology	Spontaneous initiation of atrial fibrillation by ectopic beats originating in the pulmonary veins.
Clopidogrel in Unstable Angina to Prevent Recurrent Events Trial Investigators	4566	269	Cardiology	Effects of clopidogrel in addition to aspirin in patients with acute coronary syndromes without ST-segment elevation.
Brenner BM	4538	267	Cardiology	Effects of losartan on renal and cardiovascular outcomes in patients with type 2 diabetes and nephropathy.
Hulley S	4529	226	Cardiology	Randomized trial of estrogen plus progestin for secondary prevention of coronary heart disease in postmenopausal women.
Mathers CD	4494	375	Public Health	Projections of global mortality and burden of disease from 2002 to 2030.
Haffner SM	4486	224	Cardiology	Mortality from coronary heart disease in subjects with type 2 diabetes and in nondiabetic subjects with and without prior myocardial infarction..
Flegal KM	4482	280	Public Health	Prevalence and trends in obesity among US adults, 1999-2000.
Eisenberg DM	4448	222	Alterntive Medicine	Trends in alternative medicine use in the United States, 1990-1997: results of a follow-up national survey.
Ford ES	4448	278	Endocrinology	Prevalence of the metabolic syndrome among US adults: findings from the third National Health and Nutrition Examination Survey.
Alberti KG	4448	342	Endocrinology	The metabolic syndrome--a new worldwide definition.
Levy D	4443	159	Cardiology	Prognostic implications of echocardiographically determined left ventricular mass in the Framingham Heart Study.
Murray CJ	4325	206	Public Health	Alternative projections of mortality and disability by cause 1990-2020: Global Burden of Disease Study.
Chapman PB	4317	617	Oncology	Improved survival with vemurafenib in melanoma with BRAF V600E mutation..
Calle EE	4301	287	Oncology	Overweight, obesity, and mortality from cancer in a prospectively studied cohort of U.S. adults.
Early Breast Cancer Trialists' Collaborative Group (EBCTCG)	4296	330	Oncology	Effects of chemotherapy and hormonal therapy for early breast cancer on recurrence and 15-year survival: an overview of the randomised trials.
Ogden CL	4291	1073	Public Health	Prevalence of childhood and adult obesity in the United States, 2011-2012.
Cholesterol Treatment Trialists' (CTT) Collaborators	4289	330	Biochemistry	Efficacy and safety of cholesterol-lowering treatment: prospective meta-analysis of data from 90,056 participants in 14 randomised trials of statins.
Schulz KF	4256	185	Quality of Reporting	Empirical evidence of bias. Dimensions of methodological quality associated with estimates of treatment effects in controlled trials.
Mazzaferro V	4255	193	Oncology	Liver transplantation for the treatment of small hepatocellular carcinomas in patients with cirrhosis.
Shepherd FA	4250	327	Oncology	Erlotinib in previously treated non-small-cell lung cancer.
Action to Control Cardiovascular Risk in Diabetes Study Group	4183	418	Endocrinology	Effects of intensive glucose lowering in type 2 diabetes.
North American Symptomatic Carotid Endarterectomy Trial Collaborators	4181	155	Cardiology	Beneficial effect of carotid endarterectomy in symptomatic patients with high-grade carotid stenosis.
CAPRIE Steering Committee	4177	190	Cardiology	A randomised, blinded, trial of clopidogrel versus aspirin in patients at risk of ischaemic events (CAPRIE). CAPRIE Steering Committee.
Balkwill F	4174	246	Oncology	Inflammation and cancer: back to Virchow?.
Pope CA 3rd	4163	260	Public Health	Lung cancer, cardiopulmonary mortality, and long-term exposure to fine particulate air pollution.
Spitzer RL	4139	218	Psychiatry	Validation and utility of a self-report version of PRIME-MD - The PHQ primary care study.
van de Vijver MJ	4127	258	Oncology	A gene-expression signature as a predictor of survival in breast cancer.
Hansson L	4124	206	Cardiology	Effects of intensive blood-pressure lowering and low-dose aspirin in patients with hypertension: principal results of the Hypertension Optimal Treatment (HOT) randomised trial.
Lewis EJ	4114	164	Nephrology	The effect of angiotensin-converting-enzyme inhibition on diabetic nephropathy.
Ridker PM	4110	196	Cardiology	Inflammation, aspirin, and the risk of cardiovascular disease in apparently healthy men.
Moss AJ	4110	257	Cardiology	Prophylactic implantation of a defibrillator in patients with myocardial infarction and reduced ejection fraction.
Ridker PM	4109	228	Cardiology	C-reactive protein and other markers of inflammation in the prediction of cardiovascular disease in women.
Sandler A	4095	341	Oncology	Paclitaxel-carboplatin alone or with bevacizumab for non-small-cell lung cancer.
Flegal KM	4083	510	Public Health	Prevalence and trends in obesity among US adults, 1999-2008.
Osterberg L	4076	314	Public Health	Adherence to medication.
Bernard GR	4042	238	Intensive Care	Efficacy and safety of recombinant human activated protein C for severe sepsis.
Murray CJ	4022	670	Public Health	Disability-adjusted life years (DALYs) for 291 diseases and injuries in 21 regions, 1990-2010: a systematic analysis for the Global Burden of Disease Study 2010.
Randle PJ	4021	73	Endocrinology	The glucose fatty-acid cycle. Its role in insulin sensitivity and the metabolic disturbances of diabetes mellitus.

**Table 2:** Citation Classics Journal

Journal full name	Articles	Citations	Density	Quartile (Impact Factor)	Country
New England Journal of Medicine	*57 Articles* [32 Level II] [15 Level V] [7 Level IV] [3 Level III]	*324230* [180036] [93592] [34855] [15747]	*20129* [12600] [4961] [1893] [675]	Q1 (79.260)	USA
Lancet	*21 Articles* [10 Level II] [5 Level V] [3 Level I] [1 Level III] [1 Level IV]	*137413* [82961] [30461] [13960] [4325] [5706]	*7946* 5275 1061 996 206 408	Q1 (53.254)	UK
Journal of The American Medical Association	*17 Articles* [8 Level II] [4 Level V] [2 Level III] [2 Level IV] [1 Level I]	*102392* [42245] [37119] [8430] [10342] [4256]	*6955* [2926] [2180] [1291] [373] [185]	Q1 (47.661)	UK
British Medical Journal	*4 Articles* [3 Level V] [1 Level I]	*49348* [29920] [19428]	*4358* [3433] [9925]	Q1 (23.562)	USA
Plos Medicine	*1 Article* [1 Level III]	*4494*	*375*	Q1 (11.675)	USA

**Table 3:** The coefficients of association between age and citation number/density, across various levels of evidence

	Citations~Age				Density~Age			
Level of evidence	Pearson	P-Value	Kendall	P-Value	Pearson	P-Value	Kendall	P-Value
I	0.456	0.4402	0.105	0.8005	0.266	0.6658	0.111	0.7947
II	0.391	0.005	0.0833	0.4053	-0.540	<0.001	-0.557	<0.001
III	0.494	0.2594	0.488	0.1287	-0.887	0.0078	-0.586	0.0683
IV	0.249	0.4872	-0.0227	0.9282	-0.700	0.0241	-0.796	0.0016
V	-0.235	0.228	0.0267	0.8432	-0.540	0.003	-0.709	0.0<0.001

**Figure 3:**
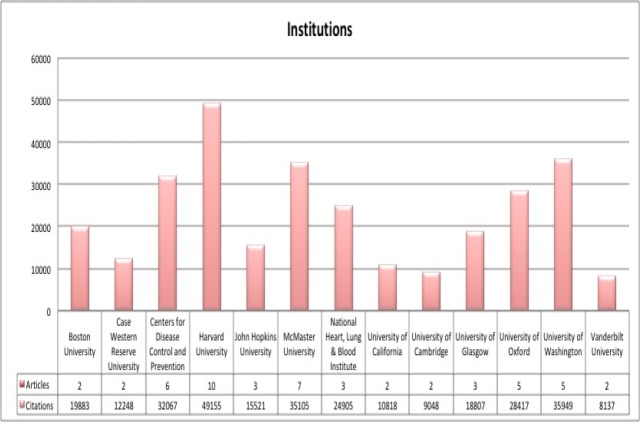
A column chart illustrating Institutions producing more than one highly cited article

The highly cited articles originated from a total of 61 institutions. Of the 61 institutions, 13 institutions had two or more of their articles appearing within the citation classics list. Amongst them, the leading institutions were Harvard University (10 articles), McMaster University (7 articles), and Centres for Disease Control and Prevention (6 articles). (Table 1, Figure 2, Figure 3).

**Citation classics’ classification and fields of medicine**

Amongst the articles extracted, 87% were original articles and the remaining 13% were review articles. All 87 original articles were published in 4 journals led by New England Journal of Medicine (53 articles), Lancet (20 articles), Journal of The American Medical Assciation (13 articles), and Plos Medicine (1 article). 

**Figure 4:**
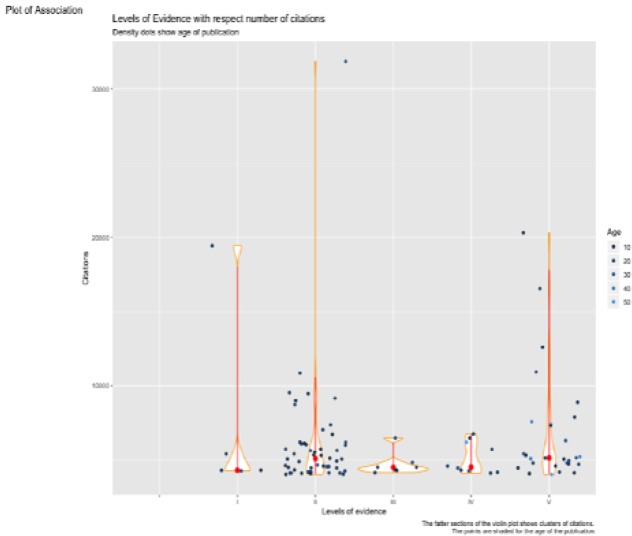
Violin plot of level of evidence with the number of citations, measured in conjunction with the age of the publication

On the other hand, review articles were published in 4 journals, called the Journal of The American Medical Association (4 articles), New England Journal of Medicine (4 articles), British Medical Journal (4 articles), and Lancet (4 articles). The number of citations for original articles ranged from 4021 to 31853 (mean = 6046). The numbers for review articles ranged from 4174 to 19428 (mean = 7065). 

The articles focused on 19 different fields. The field of cardiology was the most common speciality topic with 28 articles (167256 citations). Articles discussing oncology (19 articles, 105574 citations), public health (13 articles, 60206 citations), endocrinology (10 articles, 69400 citations) and quality of reporting (6 articles, 62455 citations) were also represented. The lowest two cited fields were radiology (4713 citations) and alternative medicine (4448 citations). Further topics of interest are listed in Table 1 and Table 2. 

**Levels of evidence and methodologies**

The 100 papers had a wide range of evidence levels. There were 50 studies at level II, 28 at level V, 10 at level IV, 7 at level III and 5 at Level I. The level of evidence I received the highest overall citation number of 305242 citations, followed by level II at 191092 citations, Level III at 50903 citations, level IV at 37644 citations, and level V at 32996 citations. For each level of evidence, there were large proportions of publications that were young (age less than 30 years old) and had less than 5000 citations. Furthermore, the level of evidence II included publications with the maximum number of citations, being under the age of 30. Likewise, level I and V also included publications with a large number of citations, with ages under 30. Level IV and V were the only ones with publications still receiving citations while being over 40 years old. These associations formed skew relationships and hence the median was used as the measure of central tendency, which can be seen as the red dot. The red dots have been placed near the centre of the fastest section of each plot (Figure 4). 

**Figure 5:**
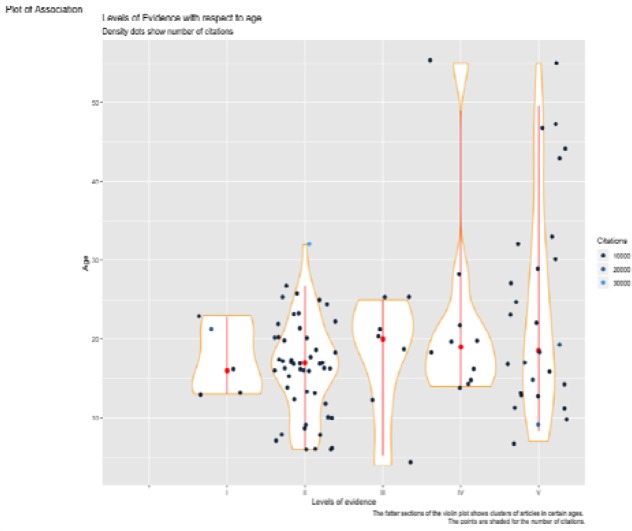
Violin plot age with the number of citations

Of the articles published in the 1960’s, 1 article was of level I and 1 was of level V. All 4 articles published in the 1970’s were of level V. During the 1990’s, authors published 15 level II articles, 5 level III, 5 level V, 4 level IV, and 2 level I articles. The decade 2000’s received contributions from 34 level II articles, 14 level V, 5 level IV, 2 level III, and 3 level I. Interestingly, for level I and IV, there are no publications younger than 12 years. On the other hand, for levels II, III and V we have publications as young as 4 years old. As seen, it is only level II that has publications with citations at 30000 citations. From the plots, it seems that 12-30 years ago, there were a great number of publications published with level of evidence II (Figure 5).

**Figure 6:**
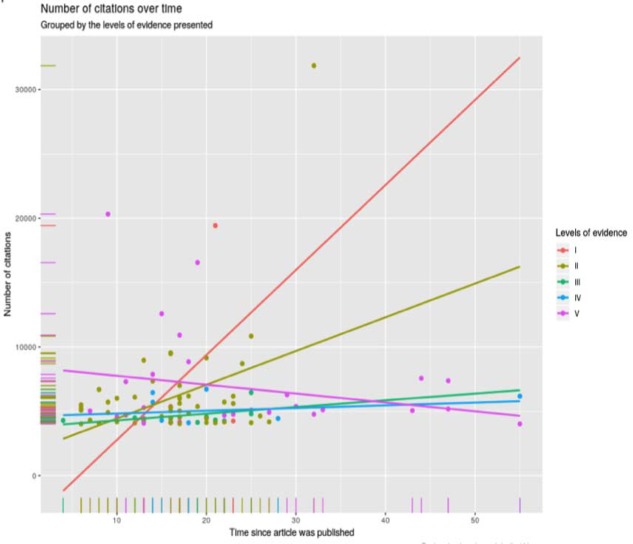
Citations over time, across the five levels of evidence

A high proportion of the published literature were RCT’s (39 articles/230004 citations), followed by cross-sectional studies (16 articles/102830 citations), systematic reviews (14 articles/90176 citations), clinical consensus (10 articles/69072 citations), case series (8 articles/59877 citations), experimental studies (8 articles/43510 citations), case-control (2 articles/16422 citations), cohort (2 articles/10592 citations) with the remainder being expert opinion (1 article/5186 citations) (Table 1).

**Statistical associations between level of evidence, citation number, density, and age**

For any level of evidence, the Shapiro-Wilk test for the assessment of normality revealed normally distributed data for citations (p>0.05) and non-normally distributed data for density (p<0.05). The Kruskal-Wallis test indicated no significant difference in the distribution of the variables; citations (p=0.3936), density (p=0.1637), and age (p=0.2904), across each level of evidence. 

-Citations over time, across all levels of evidence:

Most of the citations, across all levels of evidence, were clustered within 0-30 years old and 0-10000 citations. For level of evidence I, there was a likelihood of an increase in citations as the publication aged. Levels II, III and IV showed less considerable increase in citations over time. Level V articles exhibited a decline in the number of citations following the time of publication (Figure 6). 

**Figure 7:**
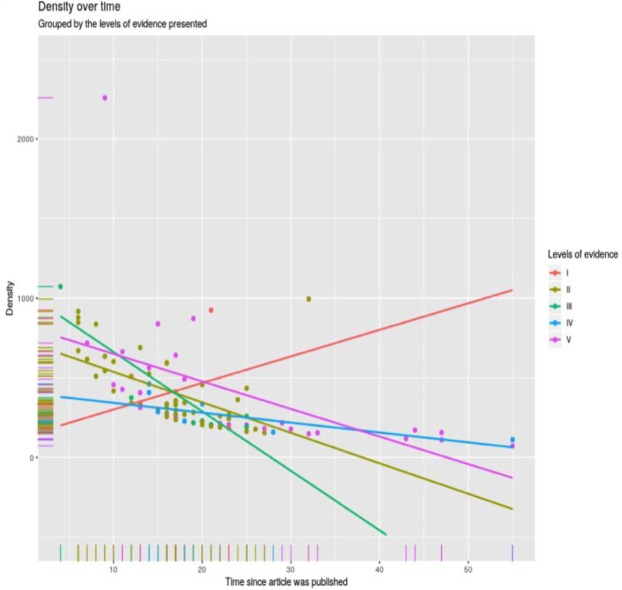
Density over time, across the five levels of evidence

-Density over time, across all levels of evidence:

There was a cluster of all types of evidence at around 0-30 years old and 0-10000 citations. Level I evidence demonstrated an increase in citation density over time. On the other hand, there was a reduction in the density over time across the remaining levels of evidence (Table 1, Table 2, Figure 7). 

Based on the plots of citations and density over age, we can make assumptions about the type of evidence presented and the effect these articles have on the field. However, since measures for citations were closer to 0 than to 1, we cannot be sure if the type of evidence is indicative of the impact (citation number).

## Discussion

Citation analysis generates a large body of statistical material, providing an insight into scientific trends and sociological diversity. In this study, we used citation indices to assess the quality of published articles. Indeed, they are tools for evaluation, however it remain an issue for further research to determine further whether evaluation tools are needed (13).

Although most scientific papers usually reach their optimum citation rate within 3 years of publication, citation indices are time-specific (14). Our examination of citation classics, published in general medical journals, demonstrated seminal contributions that were transiently popular topics. While these older seminal articles would expectedly receive a greater number of citations than more recent articles, 89% of the so-called best sellers had been published in the 1990’s and the 2000’s. This may be due to the fact that key concepts become universally accepted, and as a result, are no longer cited. 

The Journal Impact actor (JIF) reflects the citation rate of the average published article, in a particular journal, over two years (15). The five journals in which the citation classics were published were all within the top quartile. The distribution of citations across articles within a journal is not uniform. Thus, most citations within a journal come from a minority of the published articles. In spite of the weak relationship between JIF and article citation counts, editors-in chief of high impact journals tend to accept high quality articles to maintain and develop their journal’s profile and reputation. This implies that the JIF is a useful tool in assessing the quality of published articles. 

Most authors of the top-cited papers are established leaders in their field.

 Robert Merton observed that better-known scientists tend to receive more credit than their less well-known counterparts for the same achievements. Thus, authors with a significant reputation and record of publishing are cited more readily. This may be due to the fact that well-established authors tend to write quality papers and tend to present them in highly ranking journals, which are widely distributed and indexed by major abstracting services (16).

The citations classics originated from 61 institutions in 14 countries and covered 19 disciplines. The number of citations that different countries, institutions and disciplines accrue depend on factors other than quality and originality. It is well established that the citation rate varies across disciplines (14). Disciplines with longer turnover times are the most affected by the time lag. This, in turn, can also affect various institutions and countries (17, 18). The time-associated variations constitute only a part of the citation patterns. Categories, with more published articles and funding, tend to receive more citations. The scope of the discipline might be another factor that accounts for the difference. For instance, many scientists outside the field of cardiology may be citing cardiology papers. This leads to an increased number of citations beyond what we expect based on bibliometric indicators (the field cardiology constituted 28% of the citation classics). The citation pattern in other fields, such as in radiology and alternative medicine (fields with the lowest ranking citation classics), is significantly narrower as only the people in these fields cite one another (19).

One sociological aspect included the language bias. It was noted that non-English peer-reviewed materials did not make it into the list of citation classics. Citing non-English or non-Roman script papers is uncommon. A journal editor once stated ”what is useful to readers, who may want to find, read and even translate referenced articles“. We would like to raise some questions for the readers of this article - Would an article, with reference to non-English articles, be rejected by certain journals just because it has cited non-English articles? Would editors and reviewers of certain journals ask for those references to be excluded just because they could not find a reviewer who can verify them? Although English is now considered as an international language for science, data are nowadays easily accessible in the non-English literature and credit should also be given to our fellow non-English-speaking world when deemed necessary (20).

Of the citation classics, 85% had three or more authors. In applied citation analysis, multi-authored papers are generally more highly cited compared to single-authored papers. This may be due to multi-authored articles attracting a variety of practical and intellectual proficiencies and thus presenting a greater diversity of ideas and data sets (21).

Certain types of articles are bound to be cited more frequently. It is well recognised that various study designs correspond to different levels of evidence, with systematic reviews, meta-analysis, and RCT`s providing the highest quality of evidence, and case reports and expert opinions offering the lowest quality of evidence (4). Amongst the highly cited articles, clinical studies (92 articles) were far more common compared to pre-clinical (8 articles) studies. Conforming to the classification, schemed by the Oxford Centre for Evidence-Based Medicine, most articles had a high level of evidence. The levels of evidence I and II constituted almost 50% of the total number of citations gained by all articles. On the other hand, the levels IV and V constituted only 11% of the total number of citations. The regression lines in this study revealed that a paper, of level of evidence I or II, will experience a strong positive increase in citations against the age of the article. A weaker positive correlation has been noted for both levels III and IV. An article of level V on the other hand will experience a decline in citations against age. The mean yearly citation rate has increased remarkably for articles of level I. 

We suggest that the high representation of high level of evidence studies does not imply that they are the only studies being performed; rather that they have been cited more frequently compared to low level of evidence articles. For instance, in line with the plots, the last 30 years have experienced an increase in the number of published level II studies (RCT´s). This might be attributed to the improved methodological qualities of protocols and the increasing rate of technological innovation, allowing for more randomised comparisons. This has led RCT´s to become a reliable and robust source of evidence in healthcare interventions, and thus receive more popularity amongst researchers (22).

Our recommendations: 

1-The ISI should be the definitive scientific citation indexing service since it lists a large fraction of all published articles, covering broad disciplines and individual specialities. The ISI indexing service also facilitates comparisons, bibliographic coupling (identification of related articles based on common citations) and identification of co-citation studies (studies cited together in later articles) (7, 23, 24). Furthermore, the ISI can also be used to eliminate self-citations from a citation count. 

2-This study revealed that citation-based indicators are very useful, in assessing the quality of published articles, but they should be deployed, in more nuanced and open ways, alongside other metrics. 

3-Institutes should consider including citation count as part of the evaluation, for determining research priorities, allocating funding, deciding tenures, promotions and appointments, and lowering the productivity threshold, to put more focus on the quality of published work.

4-This study indicated that including self-citations in the citation count is currently a minor problem when used as a proxy for importance or quality. However, it would be worthwhile to report self-citations alongside other metrics to identify and curb excessive self-citations in the future and flag potential self-promoters.

5-Those manipulating the peer-review process to amass citations to his/her own work should be identified and removed from the editorial board or banned as reviewers. 

6-Editors should avoid artificially boosting impact factors, by encouraging the citation of a journal’s own papers. 

**limitations of this study**

The citation classics list may be criticized on a few accounts. By including articles published in general medical journals and not including subspecialty journals, several highly cited articles have been excluded from the list (25-29). Furthermore, the ISI has been reported to sporadically miss citations older than 1980. The indexing system has also been shown to have discrepancies, compared to the original publication, in at least one data field amongst 10% of the published articles (30). In the case of discrepancies, the original publication’s data were extracted. 

This study provided an insight into trends of previously published work and may serve as a useful guidance to researchers and funding bodies in evaluating the impact and assumed quality of research articles. The list of classics included in this study have influenced many people and brought major advances in the field of medicine to our attention. As Eugene Garfield, the founder of ISI, once said ”We tend to remember those works that receive the greatest public recognition“ (7). Evidence-based medicine remains the guiding principle. From this point forward, citation indices may offer us a new aspect of evidence-based medicine and arm us with extensive data that will guide our clinical decisions.

## Conflict of interests

The authors declare that they have no conflict of interest.
